# Benefits of Flexible Prioritization in Working Memory Can Arise Without Costs

**DOI:** 10.1037/xhp0000449

**Published:** 2017-08-17

**Authors:** Nicholas E. Myers, Sammi R. Chekroud, Mark G. Stokes, Anna C. Nobre

**Affiliations:** 1 Department of Experimental Psychology and Oxford Centre for Human Brain Activity, University of Oxford

**Keywords:** attention, retro-cue, visual working memory

## Abstract

Most recent models conceptualize working memory (WM) as a continuous resource, divided up according to task demands. When an increasing number of items need to be remembered, each item receives a smaller chunk of the memory resource. These models predict that the allocation of attention to high-priority WM items during the retention interval should be a zero-sum game: improvements in remembering cued items come at the expense of uncued items because resources are dynamically transferred from uncued to cued representations. The current study provides empirical data challenging this model. Four precision retrocueing WM experiments assessed cued and uncued items on every trial. This permitted a test for trade-off of the memory resource. We found no evidence for trade-offs in memory across trials. Moreover, robust improvements in WM performance for cued items came at little or no cost to uncued items that were probed afterward, thereby increasing the net capacity of WM relative to neutral cueing conditions. An alternative mechanism of prioritization proposes that cued items are transferred into a privileged state within a response-gating bottleneck, in which an item uniquely controls upcoming behavior. We found evidence consistent with this alternative. When an uncued item was probed first, report of its orientation was biased away from the cued orientation to be subsequently reported. We interpret this bias as competition for behavioral control in the output-driving bottleneck. Other items in WM did not bias each other, making this result difficult to explain with a shared resource model.

## Public Significance Statement

This study challenges the dominant model for how we remember and prioritize pieces of information over short intervals (working memory). The dominant view is that all items in working memory share a single resource, and that we can prioritize one item by redistributing resources in its favor. This view predicts that nonprioritized memories become lost or impoverished. By testing how well participants remember both prioritized and nonprioritized items, we show that this is not the case. Our findings suggest that memories can be prioritized flexibly without necessarily jeopardizing others that may still become relevant.

A prevailing account of the architecture of working memory posits that capacity is constrained by a continuous mnemonic resource that is shared among all representations (Bays, 2015; Bays & Husain, 2008; Luck & Vogel, 1997; Ma, Husain, & Bays, 2014; Matthey, Bays, & Dayan, 2015; van den Berg, Shin, Chou, George, & Ma, 2012). These capacity limits can be at least partly overcome by retrospective attention cues (‘retrocues’) presented during the delay period (Griffin & Nobre, 2003; Landman, Spekreijse, & Lamme, 2003). Resource-based models provide a potentially elegant explanation of cueing benefits: Cueing leads to a transfer of resources to the prioritized representation. This implies that uncued items are left with less of the resource and are remembered less accurately (Pertzov, Bays, Joseph, & Husain, 2013). Indeed, many studies have shown costs for invalid retrocues (Astle, Summerfield, Griffin, & Nobre, 2012; Chun, Golomb, & Turk-Browne, 2011; Gözenman, Tanoue, Metoyer, & Berryhil, 2014; Griffin & Nobre, 2003; Pertzov et al., 2013; but see Berryhill, Richmond, Shay, & Olson, 2012). This has been taken as evidence that retrocues can sharpen the mnemonic representation itself (Ma et al., 2014; Zokaei, Manohar, Husain, & Feredoes, 2014).

However, not all experiments find significant costs for uncued items. For example, conflicting evidence comes from multiple-cueing paradigms (Landman et al., 2003; Li & Saiki, 2014; Matsukura, Luck, & Vecera, 2007; Maxcey-Richard & Hollingworth, 2013; Rerko & Oberauer, 2013; van Moorselaar, Olivers, et al., 2015; see also Lepsien & Nobre, 2007). In these experiments, a first cue indicates a to-be-prioritized item, but is sometimes superseded by a second cue toward another, previously uncued, item in memory. Redirecting attention with a second cue can lead to performance improvements on a par with single-cue benefits (Rerko & Oberauer, 2013). Such results seem to argue against models of WM where cueing one item comes at a necessary and permanent cost to other items in memory.

This contradiction in the literature seems puzzling. One potential factor leading to conflicting results is that anticipation of the correct probe location or feature may at least partially explain performance improvements in multiple-cueing studies (Stokes, 2011; Makovski, Sussman, & Jiang, 2008). Another factor could relate to different strategic use of cues. Single-cue studies may encourage participants to forget uncued items (Kuo, Stokes, & Nobre, 2012; Williams, Hong, Kang, Carlisle, & Woodman, 2013), whereas multiple-cue studies may encourage a more cautious strategy in which cued items are focused but uncued items are retained (see also Souza & Oberauer, 2016). Therefore, it remains unclear whether retrocues can actually improve the representation of memory items themselves without related, compensatory costs to uncued items.

Alternatives to resource redistribution have been put forward in the literature. One prominent proposal is that retrocues confer benefits by placing the cued item in a privileged state such as the focus of attention (Griffin & Nobre, 2003; LaRocque, Lewis-Peacock, & Postle, 2014; Olivers, Peters, Houtkamp, & Roelfsema, 2011; Rerko & Oberauer, 2013; Rerko, Souza, & Oberauer, 2014; Zokaei et al., 2014). A state-change mechanism is compatible with results suggesting that the cued item is made more accessible to recall, or for guiding behavior, by occupying an *output-driving* state (Chatham & Badre, 2015; Chatham, Frank, & Badre, 2014; Myers, Walther, Wallis, Stokes, & Nobre, 2015; Wallis, Stokes, Cousijn, Woolrich, & Nobre, 2015). Under this explanation, cueing improves memory without requiring a redistribution of memory resources: the *mnemonic* representation of the cued item is not changed. Consequently, there is no reason to expect a decrease in the quality of representation of uncued items. In principle, recalling them should also carry no large costs (in agreement with the double-cueing studies), as long as probe anticipation is controlled.

To investigate what retrospective attention can reveal about WM architecture, and to address the apparent discrepancy in the literature, it is necessary to probe cued and uncued items while controlling for probe anticipation. In the current study, we probed how well cued and uncued WM items could be retrieved in four double-probe experiments. Observers encoded four oriented bars into WM. During the retention interval, either a spatial cue highlighted one memorized item or an uninformative cue provided no information about the items to be probed. Observers then recalled, in sequence, the orientation of two of the four items. On neutral-cue trials, a random two of the four items were probed. On spatial-cue trials, the cued item was always probed, along with one of the other three items. On half of spatial-cue trials, the cued item was probed first, followed by a probe to one of the three remaining items (‘cued-first’ trials). On the other half, the cued item was probed second, after an uncued item had been probed (‘uncued-first’ trials). Critically, the comparison of the second response on neutral versus cued-first trials allowed us to compare memory for uncued versus neutral items while controlling for probe anticipation. This was because, in both conditions, after making response 1 (to a neutral item on neutral trials, to the cued item on cued-first trials), participants expected one of three remaining items to be probed. In other words, probe anticipation before the second probe was identical between these two conditions. Therefore, responses to uncued items on the second probe, when compared with neutral-cue trials, would reveal any costs accrued by focusing attention on another item, but would be independent of probe expectation or response preparation. By contrast, costs to uncued items during the first probe could still be explained by expectation effects: because recall of the cued item is expected by default, unexpectedly probing the uncued item first leads to recall costs. In brief, across three experiments we found little evidence for behavioral costs suggestive of trade-offs of resource allocation when probe expectation was controlled, in spite of robust benefits in reporting the cued item.

This demonstrates that cueing benefits can, in principle, occur without sizable or correlated costs when the uncued item is recalled last. We conclude that it is possible to improve memory for a cued item without withdrawing mnemonic resources from uncued items. This result is more consistent with the alternative proposal that cued representations are transferred into an output-driving state. In addition, although the mnemonic representation of uncued items may be unharmed, we reasoned that if a retrocue primes a representation to drive the next behavior, it might subtly influence responses when a different item is unexpectedly probed. Intriguingly, the recall of uncued items was in fact biased away from the cued orientation.

## Experiment 1

### Method

#### Participants

The experimental procedures were approved by the Central University Research Ethics Committee of the University of Oxford. Twenty-four observers (16 females, mean age 23.4 years, range 18–35 years) performed a precision visual working memory task. All had normal or corrected-to-normal vision. Participants provided written, informed consent in accordance with the University ethics guidelines, and were compensated at a rate of £10 per hour. Data from three additional participants (not included in the above sample) were excluded because of poor performance (Rayleigh test on response distribution, *p* > .01).

#### Apparatus

The task was created using the Psychophysics Toolbox (Brainard, 1997) and custom scripts written in Matlab (The Mathworks). Participants sat in a dimly lit, sound-attenuated booth, at a distance of 74 cm from the monitor (22-inch Samsung Syncmaster, 1680 × 1050 pixels, 60-Hz refresh rate, 47-cm screen width). Binocular gaze locations were monitored online using an infrared video-based eyetracker (Eyelink 1000, SR Research) sampling at 500 Hz. A head rest was used to reduce head motion and improve the accuracy of the eyetracker. Participants were instructed to maintain fixation throughout the trial, except during the response and intertrial periods. Responses were made using a mouse.

#### Task

**Figure 1 fig1:**
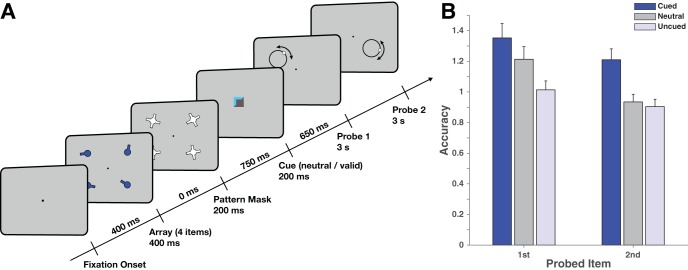
(A) Trial structure for Experiment 1. Each trial began with the appearance of a central fixation dot. After 400 ms, an array of 4 oriented bars appeared for 400 ms, followed immediately by pattern masks for 200 ms. Observers were instructed to remember the orientations of all stimuli. A cue appeared 750 ms later (for 200 ms). On retrocue trials (2/3 of all trials), the cue indicated one of the two items that would be probed (by pointing to the location where that item had been presented). Uninformative (neutral) cues appeared in the remaining 1/3 of trials. After a further delay (650 ms) the first probe appeared (a black outline in the location of one of the presented items), prompting observers to recall that item’s orientation. Observers had 3 s in which to respond to the first probe. After the full 3 s, a second item was probed, with the same time constraint. After responding about both items, observers saw the correct orientations superimposed over their response orientations for the two probed items (not shown). (B) Behavioral performance in Experiment 1. Recall accuracy (1/*SD*) was modulated both by cue type and by probe order. Error bars denote within-observer standard error of the mean.

On each trial, observers had to remember the orientations of four peripherally presented bars (see Figure 1). Two of these were then probed in sequence. On neutral-cue trials, these items were randomly selected. On retrocue trials, one of the two probed items was cued during the delay. Orientations were retrieved by means of continuous recall estimates. The experiment involved three blocks of practice and eight blocks of the main task. Each block consisted of 60 trials (20 neutral, 20 cued item probed first, 20 cued item probed last), resulting in a total task length of 480 trials.[Fig-anchor fig1]

On each trial, participants saw a stimulus array (presented for 400 ms) consisting of four oriented bars around a central fixation dot (a black square with 0.20° visual angle edge length, on a gray background). Oriented bars (2.60° × 0.30°, with a disk of 0.70° diameter at one end to denote the axis of rotation) appeared at the center of each quadrant, at a radial eccentricity of 5° from fixation. Stimuli were immediately masked (for 200 ms) to minimize the contribution of iconic memory on performance. Masks consisted of randomly oriented white crosses (created by overlapping two oriented bars at 90° from one another). After a delay of 750 ms, a central cue appeared for 200 ms (0.40° edge length). Neutral cues (1/3 of trials) were black squares. Retrocues (2/3 of trials) contained a colored corner (blue), indicating which of the four items would be probed. After a 650-ms delay, a probe appeared at a previous stimulus location.

Probes consisted of a black circle with a radius equal to the length of the bar. After participants started moving the mouse to respond to the orientation, a small tracking disk appeared at a random location on the circle. This probe design was intended to minimize interference from the probe on memory recall because the only orientation-specific part of the probe—the tracking disk—appeared after participants started moving the mouse, and presumably after they had recalled the orientation from memory. Likewise, because the black probe circle lacked any orientation-specific features, it minimized any strategic benefits of probe anticipation (Makovski et al., 2008). Participants were instructed to move the mouse to rotate the tracking disk to the remembered orientation of the bar at the probed location. They locked in the current orientation with a mouse click. Observers had a 3,000-ms window in which to respond. The first probe remained on the screen for 3,000 ms, irrespective of response time, to avoid any speed–accuracy trade-off between responses to successive items.

#### Analysis

For each response, we calculated the deviation (in degrees) of the recalled orientation from the actual orientation of the probed stimulus, generating a distribution across trials. Recall accuracy was defined as the inverse of the circular standard deviation of the response distribution (Bays, Catalao, & Husain, 2009), separately for each condition. Repeated-measures analysis of variance with the factors cue (neutral, cued, and uncued) and response (first, second) was used to compare accuracy between conditions. Benefits and costs were also established separately by comparing only cued and neutral or uncued and neutral trials. This was a crucial analysis to establish, especially for the second response, whether uncued items suffered a cost.

We also fit a mixture of two distributions to observers’ responses:

$$\text{p}\left(\text{x|θ}\right)={\text{ P}}_{\text{MEM}}\text{×}\text{VonMises}\left(\text{x},0,\text{κ}\right)+\left(1-{\text{P}}_{\text{MEM}}\right)\frac{1}{2\text{π}}\;,$$

where *x* is the response angle, θ is the presented angle, *VonMises* denotes the von Mises distribution, that is, the circular analogue of the normal distribution, and (1 − P_MEM_)/2π is the height of the uniform distribution arising form randomly distributed guesses. The two variable parameters, P_MEM_ and κ, define the two distributions: the probability of recalling an item (P_MEM_), and the concentration parameter, or precision, of remembered items (κ). This model was fit using maximum-likelihood estimation (MLE, Myung, 2003), separately for each condition. Model parameters for different conditions were compared using ANOVA, as above. Previous studies have found that retrocues affect mainly the likelihood of recall (P_MEM_), having little or no effect on the precision (κ) of memory representations (Murray, Walther, Wallis, Stokes, & Nobre 2013; Wallis et al., 2015; but see Williams et al., 2013). We therefore focused on how cues changed the recall rate.

Because we were particularly interested in potential null effects (i.e., no costs to uncued items in WM), we used Bayes Factors (BF; Dienes, 2011; Kass & Raftery, 2012) calculated from Bayesian *t* tests and Bayesian ANOVAs (Rouder, Morey, Speckman, & Province, 2012) to establish whether the lack of significance indicated a genuine absence of difference, or merely a lack of sensitivity. Analyses were performed in R (R Core Team, 2016) using the BayesFactor 0.9.12–2 package (Morey & Rouder, 2015). We estimated the likelihood of data in the light of two models: a null model (M_0_) that only assumes between-participants variability with no effect of task conditions and an alternative model (M_1_) that includes task conditions. The likelihood ratio between these models is the BF. The BF indicates how much more likely one model is than the other, given the data (and accounting for the difference in model complexity). A comparison between two models can be interpreted as a test of the factors differing between them. BFs below 3 are usually regarded as “weak” evidence, between 3 and 10 as “substantial,” between 10 and 100 as “strong” and above 100 as “decisive” evidence in favor of the model under consideration. Following this general guideline, BFs below 1/3 were interpreted as genuine null effects (i.e., “substantial” evidence in favor of the null model).

#### Trial-by-trial correlations

Trade-offs in resource allocation should lead to negative correlations in accuracy across trials. To quantify trial-by-trial correlations, we calculated Pearson correlations between absolute error for the first and second response across trials. Correlations were calculated separately for each condition, Fisher-transformed, and tested for significance with (Bayesian) ANOVAs and *t* tests.

#### Recall bias analysis

In addition to influencing recall accuracy, retrocues could also have more subtle effects on the mutual interactions between WM representations. In particular, if retrocues transfer the cued item into a state (such as the focus of attention) that has privileged access to driving the next behavior, unexpected recall of uncued information might be influenced by the cued item, even if mnemonic representations of the two items remained unchanged. For instance, recall of uncued items might be attracted toward or repulsed from the cued feature (Fischer & Whitney, 2014; Kang & Choi, 2015; Wildegger, Myers, Humphreys, & Nobre, 2015). We tested for such distortions by quantifying biases arising from the cued item during the recall of the uncued item. Trials were binned (into 64 bins, each containing 1/4 of all trials, see also Cravo, Rohenkohl, Wyart, & Nobre, 2013) according to the relative orientation of the second probed orientation with respect to the first probed orientation, separately for the three conditions (cued probed first, cued probed second, and neutral). The average response bias was calculated for each bin, yielding a bias curve as a function of relative orientation between the two angles in memory. If there was a consistent attraction effect, then response bias should be largely negative for negative relative orientations, and positive for positive relative orientations. A repulsive (orthogonalizing) effect would create the opposite pattern: a positive bias for negative relative orientations, and vice versa. To quantify whether there was any influence, the area under the bias curve was integrated, separately for negative and positive relative orientations. If the average area for positive relative orientations was larger than for negative orientations, this indicated an *attractive* effect of the unprobed stimulus, whereas a negative area difference would indicate a *repulsive* bias. Significant bias was assessed using a one-sample *t* test on the area difference. This analysis was repeated for the second response (with respect to the item probed during the first response), separately for each cue condition.

### Results

#### Accuracy

Our main outcome measure was accuracy, defined as the inverse of the circular standard deviation of the response distribution. There were robust cueing effects on accuracy (Figure 1b, 2 × 3 ANOVA, main effect of cue: *F*_2,46_ = 37.09, *p* = 2.56 × 10^−10^, partial eta squared, η_p_^2^ = 0.62, Bayes Factor, BF = 6.27 × 10^9^). Response order also had a significant effect (*F*_1,23_ = 34.22, *p* = 5.82 × 10^−6^, η_p_^2^ = 0.60, BF = 3.60 × 10^3^). In addition, there was a weak but significant interaction (*F*_2,46_ = 6.96, *p* = .0023, η_p_^2^ = 0.23, BF = 2). To examine cueing benefits separately from costs, an ANOVA was conducted using only cued and neutral responses, again showing an effect of cueing (2 × 2 ANOVA with cued and neutral responses, main effect of cue: *F*_1,23_ = 23.99, *p* = 6.01 × 10^−5^, η_p_^2^ = 0.51, BF = 1.15 × 10^3^). There was also a weak interaction with the cue effect (*F*_1,23_ = 5.66, *p* = .026, η_p_^2^ = 0.20, BF = 1.09), indicating that cueing benefits were only marginally stronger on the second (*t*_23_ = 5.36, *p* = 1.9 × 10^−5^, BF = 1.19 × 10^3^) than the first response (*t*_23_ = 2.74, *p* = .012, BF = 4.28).

Importantly, there was a strong interaction when comparing neutral to uncued items (both main effects: *F*_1,23_ > 31, *p* < 10^−5^, order-by-cue interaction *F*_1,23_ = 21.34, *p* = 1.2 × 10^−4^, η_p_^2^ = 0.48, BF = 46.5), owing to the strong cueing cost during the first response (*t*_23_ = −5.54, *p* = 1.2 × 10^−5^, BF = 1.78 × 10^3^), with only an inconclusive cost during the second response (*t*_23_ = −2.10, *p* = .047, BF = 1.36). This pattern confirmed our key prediction that costs were significantly reduced during the second response (*t*_23_ = −4.62, *p* = 1.2 × 10^−4^). However, although the cost was small, it was not conclusively absent. In the next experiment, we tested whether this could be eliminated entirely by cueing the order in which the cued and uncued item would be probed.

Previous studies have found that decomposing response errors using a mixture model (that allows for the separate measurement of response precision and the likelihood of remembering the probed item, see Bays et al., 2009; Zhang & Luck, 2008; Suchow, Brady, Fougnie, & Alvarez, 2013) can be informative in measuring the effects of retrocues (Murray et al., 2013). We replicated previous results showing that retrocues mainly improve recall rates (see Supplementary Results, Figures S1, S3, S4, and S5). Across experiments, the outcome of the mixture modeling was largely in agreement with the accuracy results reported in the main text (i.e., increased recall rate for cued items during both the first and second probe, increased precision only for cued items during the first probe, and with no impairment on recall rate or precision for uncued items during the second probe).

In addition, we found that retrocues tended to increase the precision of recall when the cued item was probed first (see Supplementary Results), but not when it was probed second. Effects on precision have been observed before (van Moorselaar, Gunseli, et al., 2014; Wallis et al., 2015), but not consistently (e.g., Murray et al., 2013).

#### Trial-by-trial correlations

It is possible that more subtle trade-offs in accuracy occurred on a trial-by-trial basis, such that trials in which the cued item was remembered better than average led to reduced accuracy for uncued items. The absolute angular error for the first and second response on each trial was used to calculate the Pearson correlation coefficient across trials, separately for each condition (neutral, cued item probed first, and cued item probed second). If there was any trade-off, there should be a negative correlation between cued and uncued accuracy in the cueing conditions. Overall, correlations were weak (average across conditions, Pearson *r*: 0.022 ± 0.012, *t* test on Fisher-transformed correlation values: *t*_23_ = 1.79, *p* = .0873, BF = 0.845). There was a consistent positive correlation when the cued item was probed first (Pearson *r*: 0.052 ± 0.022, *t*_23_ = 2.35, *p* = .0275, BF = 2.09), but not in the other two conditions (neutral: 0.011 ± 0.019, *t*_23_ = 0.57, *p* = .574, BF = 0.249, cued item probed second: 0.002 ± 0.017, *t*_23_ = 0.11, *p* = .912, BF = 0.216, main effect of cue, *F*_2,46_ = 2.103, *p* = .134, η_p_^2^ = 0.08, BF = 0.70). Therefore, there was no evidence that resource redistribution might lead to an accuracy tradeoff across trials (which would have manifested as a negative correlation, or at least as a significantly lower correlation on cued compared with neutral trials).

#### Behavioral orthogonalization of recalled items

In spite of the absence of a strong trade-off in accuracy, there might be more subtle effects of the cued item on the recall of uncued items. As outlined above, the accuracy improvement from cues in the current design is hard to explain solely via a permanently increased share of the memory resource, or via increased probe anticipation (since probe anticipation should not improve recall in our task design, see also Method). The benefit could instead (or additionally) arise because the representational state of the cued item has changed (Murray et al., 2013). One possibility is that after the cued item has been selected, it is stored in a state that can better drive behavioral output. As a result, unexpected probes for a *different* item could result in behavior that is biased by the orientation of the cued item, even if the underlying mnemonic representation of the uncued item is not changed.

**Figure 2 fig2:**
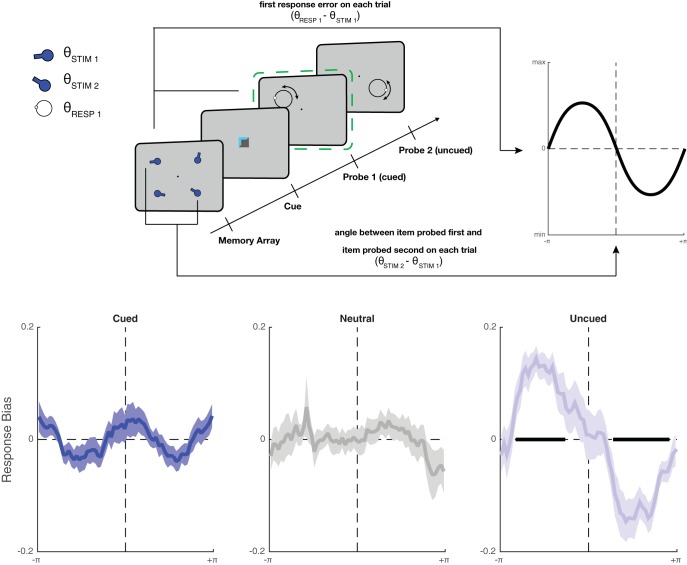
Response bias during serial probe recall in Experiment 1. Response bias (in radians) during the first of two responses, with respect to the orientation of the item that was probed second (also in radians). Each panel shows a different cueing condition, with the condition label (Cued, Neutral, Uncued) referring to the item that is being recalled. Each plot shows the average response bias (*y* axis: negative values indicate that responses were, on average, clockwise to the correct orientation of the probed item) with respect to the angular difference between the two probed orientations (*x* axis: negative values indicate that the second item’s orientation was clockwise to the currently probed orientation). Shading indicates *SEM*, and black bars indicate points on the curve that showed significant bias (*p* < .05, uncorrected, only shown for conditions with an overall significant bias, see Method). The first response was generally not biased toward or away from the second item, with one exception: when the retrocued item was probed last, it exerted a significant repulsive bias on recall of the uncued item (right panel).

This was tested in the first experiment by binning trials according to the angular difference between the orientation of the first and the second probed item. In each bin, the response bias was calculated. On trials when an uncued item was probed first (and, consequently, the cued item had to be held in mind for the second response), recall of the uncued item was significantly biased *away* from the orientation of the cued item (Figure 2, right panel, *t*_23_ = −5.12, *p* = 3.44 × 10^−5^, BF = 707). In other words, when the cued orientation was clockwise with respect to the probed orientation, responses to the probed orientation were biased in the counterclockwise direction (and vice versa). There was no such bias on neutral trials or on trials when the cued item was probed first (all *p* > .53, BF < 0.26, resulting in a main effect of cue, *F*_2,46_ = 13.18, *p* = 2.98 × 10^−5^, η_p_^2^ = 0.36, BF = 3.37 × 10^3^). Therefore, bias during the first response appeared only when the cued item had to be kept in memory for recall during the second probe.[Fig-anchor fig2]

This result establishes that cued items that have not yet been recalled can have a repulsive effect on the recall of other items. Therefore, observers may have been preparing to recall the cued item at the first response on each trial (see also Experiments 2 and 3).

Orthogonalization could arise at several processing stages. One possibility is that the effect occurs in a response-planning circuit, rather than at an earlier, perceptual or memory-maintenance stage. A reason to believe this is that the orientation of an uncued or neutral item seems not to have any repulsive effect, even though it is still held in memory. Therefore, the recall of the uncued item (rather than the representation of its visual features) could be biased away from a concurrently formed response plan (to the cued item). Furthermore, during the second response, cues had no effect on recall bias. While, overall, the second response was biased away from the first response (see also Kang & Choi, 2015), bias was not modulated by cue type (*F*_2,46_ = 2.33, *p* = .108, η_p_^2^ = 0.09, BF = 0.752, see Supplementary Results, Fig. S2). Therefore, the cued item may not have permanently biased uncued mnemonic representations, but rather influenced behavior only when it had not yet been recalled.

## Experiment 2

### Method

A second experiment attempted to replicate the findings of Experiment 1 and to assess whether knowledge of probe order had an influence on the benefits and costs of cueing (Figure 1B).

#### Participants

Twenty-four new observers (13 females, mean age 24.1 years, range 19–31 years) participated in Experiment 2. All had normal or corrected-to-normal vision. Participants provided written consent in accordance with the University ethics guidelines, and were compensated at a rate of £10 per hour. Six additional participants (not included in the sample of 24) were excluded because of poor performance (using the same criteria as in Experiment 1).

#### Task

The task was similar to Experiment 1, with the exception that retrocues also gave information about probe order. On each cue trial, the color of the cue indicated whether the cued item would be probed first or second (100% valid). Cue colors (orange and blue) were equiluminant, and were counterbalanced across participants. Participants were instructed about the cue-order mapping before the experiment started. Because of the increased demands in parsing the cue, the delay between cue offset and onset of the first probe was increased to 1,300 ms. Other timings were kept the same (with the exception of a minor increase in the delay between array offset and cue onset from 750 to 800 ms). Analyses were identical to those in Experiment 1.

### Results

#### Accuracy

**Figure 3 fig3:**
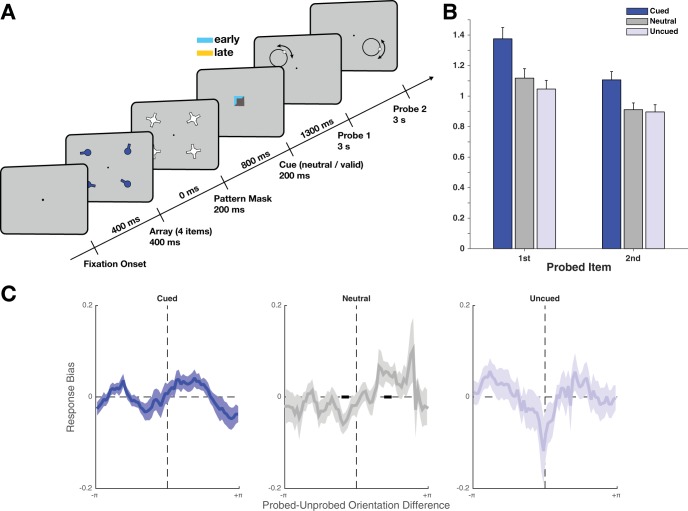
(A) Trial structure for Experiment 2. The structure was similar to Experiment 1, with the exception that the cue color indicated whether that item would be probed first or second. The delay between the cue and the first probe was longer (1,300 ms) to give observers more time to parse the more complicated cue. (B) Behavioral performance in Experiment 2. Recall accuracy was modulated both by cue type and by probe order. Importantly, during the second response, uncued items had equivalent accuracy to neutral items, indicating that there are no lingering costs to improving the accuracy of a retrocued item. Error bars denote within-observer standard error of the mean. (C) Response bias in Experiment 2 (which cued whether the retrocued item would be probed first or second), during the first response, with respect to the orientation of the item that was probed second. Recall of uncued items was not significantly biased by the cued orientation.

In Experiment 2, we tested whether additionally cueing the order in which items were recalled could further reduce cueing costs. Experiment 2 (see Figure 3A for design) broadly replicated Experiment 1. There were robust cueing effects on accuracy (Figure 3B, 2 × 3 ANOVA, main effect of cue: *F*_2,46_ = 37.28, *p* = 2.38 × 10^−10^, η_p_^2^ = 0.62, BF = 6.08 × 10^8^), as well as a significant effect of response order (*F*_1,23_ = 96.98, *p* = 1.02 × 10^−9^, η_p_^2^ = 0.81, BF = 7.90 × 10^6^), and a modest but inconclusive interaction (*F*_2,46_ = 3.60, *p* = .035, η_p_^2^ = 0.14, BF = 0.65). An ANOVA using only cued and neutral responses (to test for benefits independent of costs) also showed an effect of cueing (2 × 2 ANOVA, main effect of cue: *F*_1,23_ = 41.45, *p* = 1.43 × 10^−6^, η_p_^2^ = 0.64, BF = 4.43 × 10^4^), and also a main effect of order (*F*_1,23_ = 67.68, *p* = 2.65 × 10^−8^, η_p_^2^ = 0.75, BF = 2.12 × 10^5^). There was no cue-by-order interaction (*F*_1,23_ = 3.00, *p* = .097, η_p_^2^ = 0.12, BF = 0.443), indicating that cueing benefits were comparable on the first (*t*_23_ = 6.27, *p* = 2.15 × 10^−6^, BF = 8.87 × 10^3^) and the second response (*t*_23_ = 5.17, *p* = 3.05 × 10^−5^, BF = 790).[Fig-anchor fig3]

Analysis of cueing costs, by comparing neutral to uncued items, revealed no strong interaction when comparing neutral to uncued items (order by cue interaction *F*_1,23_ = 1.46, *p* = .239, η_p_^2^ = 0.06, BF = 0.58, main effect of cue: *F*_1,23_ = 5.77, *p* = .0247, η_p_^2^ = 0.20, BF = 0.52, main effect of order: *F*_1,23_ = 64.7, *p* = 3.9 × 10^−8^, η_p_^2^ = 0.74, BF = 6.86 × 10^8^), owing to the negligibly small cueing costs during the first response (*t*_23_ = −1.98, *p* = .060, BF = 1.13), with no cueing costs during the second response (*t*_23_ = −0.72, *p* = .482, BF = 0.27). The overall absence of cueing costs contrasts with Experiment 1. Specifically, there were no significant cueing costs during either response. This resulted in a significant cue-by-experiment interaction (*F*_1,46_ = 7.04, *p* = .011, with a trend toward a three-way interaction of cue by experiment by response, *F*_1,46_ = 3.68, *p* = .061), driven mainly by a significant reduction in the cueing costs during the first response (two-sample *t* test, *t*_46_ = −2.53, *p* = .0148, second response: *t*_40.8_ = −0.60, *p* = .552). In fact, during the second response, the Bayes factor analysis favored the interpretation that there is no difference between neutral and uncued trials. This could indicate that the order cue permitted temporal compartmentalization of prioritization, reducing the preparation of the cued item during the first probe.

#### Trial-by-trial correlations

As with Experiment 1, there was no evidence for trial-by-trial tradeoffs in accuracy. Instead, we saw modest but significantly *positive* correlations for all three cueing conditions (mean ± *SEM* Pearson *r* for neutral: 0.055 ± 0.022, *t*_23_ = 2.52, *p* = .019, BF = 2.85, cued first: 0.061 ± 0.017, *t*_23_ = 3.68, *p* = .0012, BF = 29.3, cued second: 0.059 ± 0.018, *t*_23_ = 3.26, *p* = .0035, BF = 11.9). Correlations did not diminish in magnitude on cued trials compared with neutral trials (cued first vs. neutral: *t*_23_ = 0.23, *p* = .817, BF = 0.22, cued second vs. neutral: *t*_23_ = 0.12, *p* = .902, BF = 0.216, main effect of cue, *F*_2,46_ = 0.023, *p* = .977, η_p_^2^ = 0.001, BF = 0.12). This again contradicts the hypothesis of any trade-off across trials.

#### Behavioral orthogonalization of recalled items

In Experiment 1, the biasing influence on behavior of the subsequently probed, retrocued item could have appeared because observers did not know whether the cued item would be probed first or second. Therefore, observers might have prepared to recall the cued item by default, resulting in a bias when, unexpectedly, an uncued item was probed first. In Experiment 2, the cue color indicated probe order: this might have allowed observers to compartmentalize the recall process, and to prevent the retrocued item from influencing recall when they knew that an uncued item would be probed first. In line with this prediction, there was no biasing effect in Experiment 2. Specifically, recall of the uncued item (during the first response) was unaffected by the orientation of the retrocued item (Figure 3B, *t*_23_ = −0.21, *p* = .838, BF = 0.219, two-sample *t* test comparing bias in Experiments 1 and 2, *t*_44.7_ = −3.77, *p* = 4.74 × 10^−4^). There was also no consistent biasing effect across all conditions (mean across conditions: *t*_23_ = 1.21, *p* = .239, BF = 0.410, main effect of condition: *F*_2,46_ = 2.09, *p* = .137, η_p_^2^ = 0.08, BF = 0.677), although there was an attraction effect for neutral trials (*t*_23_ = 2.33, *p* = .029, BF = 2.02). However, this modest attractive effect on neutral-cue trials was not replicated in any of the other experiments.

## Experiment 2b

Experiment 2 showed clear benefits with low or no costs. We aimed to replicate this finding in Experiment 2b. Experiment 2 differed from Experiments 1 and 3 in that the delay between the retrocue and the probe was slightly longer, to allow for additional processing time that was presumably required for parsing the additional information about probe order present in the cue. We sought to confirm that a long delay between the cue and the probe can reliably create benefits without costs. This was tested in Experiment 2b.

### Method

#### Participants

Sixteen new observers (7 females, mean age 21.4 years, range 18–30 years) participated in Experiment 4. All had normal or corrected-to-normal vision. Participants provided written consent in accordance with the University ethics guidelines, and were compensated at a rate of £10 per hour.

#### Task

**Figure 4 fig4:**
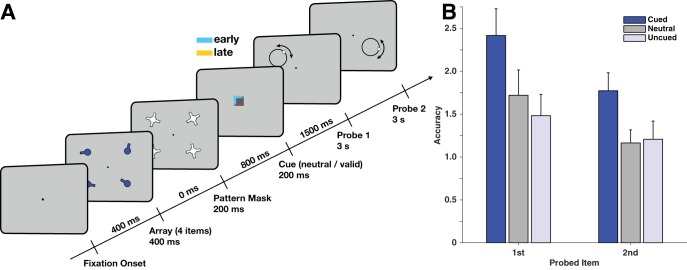
(A) Trial Structure for Experiment 2b. The structure was the same as Experiment 2, with the exception that the cue-probe delay was slightly longer (1,500 ms) to give observers more time to parse the more complicated cue. (B) Behavioral performance in Experiment 2b. Recall accuracy was modulated both by cue type and by probe order. Error bars denote within-observer standard error of the mean.

The task (Figure 4a) was similar to Experiment 2. Again, retrocues gave information about probe order in addition to which item would be probed. Cue order was also held constant for an entire block of 60 trials. We blocked cue order so that participants would make use of it more easily. Nonetheless, on each cue trial, the color of the cue also served as a reminder of whether the cued item would be probed first or second (100% valid). Cue colors were counterbalanced across participants. Participants were instructed about the color-order mapping before the experiment started. The delay between cue offset and onset of the first probe was further increased to 1,500 ms. Other timings were kept the same as in Experiment 2. Analyses were identical to those in Experiment 2.[Fig-anchor fig4]

### Results

In Experiment 2b, we tested whether the absence of cueing costs in Experiment 2 could be replicated in a design with a similarly long cue-probe delay. Experiment 2b replicated the results from Experiment 2 (see Supplementary Results and Fig. S4 for details and for orthogonalization and trialwise correlation analyses). We found a strong cueing benefit for both the first (Figure 4B, *t*_15_ = 5.775, *p* = 3.66 × 10^−5^, BF = 694) and the second response (*t*_15_ = 4.91, *p* = 1.88 × 10^−4^, BF = 164). Costs to uncued items, however, were only present during the first response (*t*_15_ = −3.71, *p* = .002, BF = 20). There were no costs during the second response, as confirmed by the low BF (*t*_15_ = 0.53, *p* = .606, BF = 0.29). The absence of cueing costs during response 2 confirms the finding from Experiment 2: robust cueing benefits can be observed at no cost to uncued items. Nevertheless, the significant costs during response 1, even when order was cued, indicate that performance on uncued items may still be worse when a different item is in a prioritized state.

In line with the accuracy results, trial-by-trial correlations also showed no evidence of tradeoffs, since they were either absent (in the cueing conditions) or slightly positive (in the neutral condition). As expected, and replicating the absence of an effect in Experiment 2, we also found no evidence for orthogonalization of the uncued item with respect to the cued item (see Supplementary Results).

## Experiment 3

### Method

Experiment 1 showed that cueing one item in memory can bias recall of other, concurrently held items. As expected, cueing the order of recall eliminated this bias (in Experiments 2 and 2b). In Experiment 3, we aimed to replicate the bias found in Experiment 1 and to evaluate within a single group whether foreknowledge of the recall order influenced this bias.

#### Participants

Twenty new observers (eight females, mean age 22.9 years, range 19–28 years) participated in Experiment 3. All had normal or corrected-to-normal vision. Participants provided written consent in accordance with the University ethics guidelines, and were compensated at a rate of £10 per hour. Three participants (not included in the sample of 20) were excluded because of poor performance (according to the same criteria as in Experiments 1 and 2).

#### Tasks

Task parameters were based on Experiment 1 (Figure 1A). Two versions of the task were completed in separate sessions separated by at least 24 hours. In one session, the task was identical to Experiment 1. In the other session, retrocues always gave information about the probe order (as in Experiment 2). Session order was counterbalanced across participants. In both sessions, the delay between cue and probe was the same as in Experiment 1. Analyses were identical to those in Experiments 1 and 2, with the addition of cue order (order shown/not shown) as a factor in the analyses of variance. Because Experiment 3 contained this additional factor, we reduced trial numbers (to 100 trials per condition, yielding a total of 600 trials per participant).

### Results

#### Behavioral orthogonalization of recalled items

**Figure 5 fig5:**
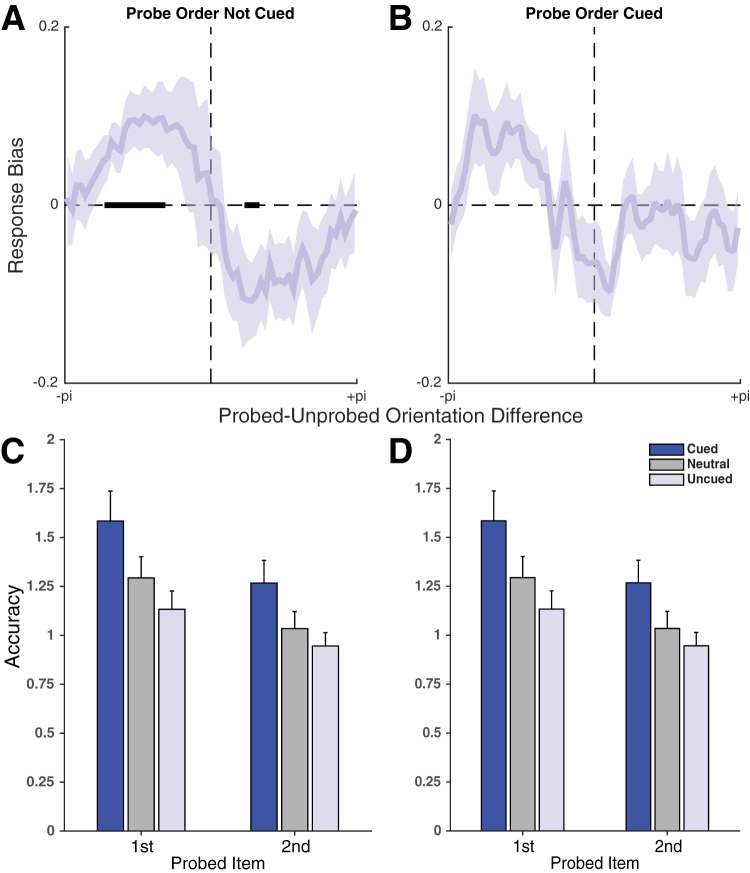
(A) Response bias for recall of uncued items in Experiment 3, during the first of two responses, with respect to the orientation of the item that was probed second. Conventions are the same as in Figure 2. When probe order was not known in advance, the results replicated those in Experiment 1: recall of an uncued item was significantly biased away from the cued item held in WM. (B) When probe order was known from the time of the retrocue as in Experiment 2, compare Figure 3c), this effect was no longer significant, confirming the finding in Experiment 2. Accuracy effects largely mirrored those of Experiments 1–2, with benefits to cued items and no significant costs when an uncued item was probed second. This pattern was present both when order was not cued (C) and when it was cued (D).

The main purpose of Experiment 3 was to replicate the significant repulsive bias found in Experiment 1. When probe order was not cued (as in Experiment 1), we saw the same bias again: Recall of the uncued item (during the first response) was biased away from the orientation of the retrocued item (Figure 5A, *t*_19_ = −2.97, *p* = .0079, BF = 6.26, resulting in a main effect of cue, *F*_2,38_ = 5.341, *p* = .009, η_p_^2^ = 0.22, BF = 10.5). By contrast, when probe order was cued (as in Experiment 2), there was no longer a significant bias, although there was a slight trend (Figure 5B, *t*_19_ = −1.67, *p* = .112, BF = 0.755, and no main effect of cue, *F*_2,38_ = 2.11, *p* = .14, η_p_^2^ = 0.10, BF = 0.803). The bias did not differ significantly between tasks on uncued responses (*t*_19_ = −0.902, *p* = .378, BF = 0.334). During the first response, the other conditions (neutral, cued) did not show a biasing effect, whether or not response order was cued (all *t* < 1.51, *p* > .147, BF < 0.612). Cues again had no significant effect on bias during the second response (see Supplementary Results).[Fig-anchor fig5]

#### Accuracy

In addition to replicating the bias effect, Experiment 3 also confirmed the accuracy results found in the first two experiments. Irrespective of whether order was cued (Figure 5C) or not (Figure 5D), we saw significant cueing benefits (see detailed results below and Supplementary Results and Figures S5 and S6). Again, we saw no significant cueing costs during the second response. During the first response, cueing costs were significant, independent of order cueing.

Cueing probe order had no effect on performance (2 × 2 × 3 ANOVA with factors order cue, probe order, and cue type, main effect of order cue: *F*_1,19_ = 0.18, *p* = .673, η_p_^2^ = 0.01, BF = 0.161, all interactions involving order cue: *F* < 1.50, *p* > .24, BF < 0.209). For an easier comparison with Experiments 1 and 2, we present results separately by order cueing condition.

#### Probe order not cued (as in E1)

There were robust cueing effects on accuracy (Figure 5C, 2 × 3 ANOVA, main effect of cue: *F*_2,38_ = 16.12, *p* = 8.54 × 10^−6^, η_p_^2^ = 0.46, BF = 9.58 × 10^4^), as well as a significant effect of response order (*F*_1,19_ = 27.19, *p* = 4.94 × 10^−5^, η_p_^2^ = 0.59, BF = 189). There was no significant interaction (*F*_2,38_ = 1.61, *p* = .214, η_p_^2^ = 0.08, BF = 0.287). An ANOVA using only cued and neutral responses showed effects of cueing (2 × 2 ANOVA with cued and neutral responses, main effect of cue: *F*_1,19_ = 14.07, *p* = .00135, η_p_^2^ = 0.43, BF = 325) and of order (*F*_1,19_ = 27.22, *p* = 4.91 × 10^−5^, η_p_^2^ = 0.59, BF = 30, no interaction with the cue effect: *F*_1,19_ = 2.12, *p* = .162, η_p_^2^ = 0.10, BF = 0.519), indicating that cueing benefits were comparable on the first (*t*_19_ = 3.12, *p* = .00568, BF = 8.24) and the second response (*t*_19_ = 3.76, *p* = .00134, BF = 28.4). There was no interaction when comparing neutral to uncued items, and a negligible effect of the cue (main effect of cue: *F*_1,19_ = 5.97, *p* = .0245, η_p_^2^ = 0.24, BF = 0.711, main effect of order: *F*_1,19_ = 14.37, *p* = .00124, η_p_^2^ = 0.43, BF = 1.89 × 10^3^, order by cue interaction *F*_1,19_ = 0.020, *p* = .890, η_p_^2^ = 0.001, BF = 0.298), owing to the comparably small cueing costs during the first response (*t*_19_ = −2.22, *p* = .0391, BF = 1.69), with no noticeable cueing costs during the second response (*t*_19_ = −1.47, *p* = .157, BF = 0.59).

#### Probe order cued (as in E2)

There were robust cueing effects on accuracy (Figure 5D, 2 × 3 ANOVA, main effect of cue: *F*_2,38_ = 20.31, *p* = 1.00 × 10^−6^, η_p_^2^ = 0.51, BF = 5.72 × 10^4^), as well as a significant effect of response order (*F*_1,19_ = 52.28, *p* = 7.27 × 10^−7^, η_p_^2^ = 0.73, BF = 4.07 × 10^4^). There was no significant interaction (*F*_2,38_ = 2.37, *p* = .107, η_p_^2^ = 0.11, BF = 0.489). An ANOVA using only cued and neutral responses showed an effect of cueing (2 × 2 ANOVA with cued and neutral responses, main effect of cue: *F*_1,19_ = 17.36, *p* = .000523, η_p_^2^ = 0.48, BF = 21.2). There was also a main effect of order (*F*_1,19_ = 46.59, *p* = 1.628 × 10^−6^, η_p_^2^ = 0.71, BF = 4.49 × 10^4^). There was no interaction with the cue effect (*F*_1,19_ < 0.01, *p* = .996, η_p_^2^ < 0.0001, BF = 0.309), indicating that cueing benefits were comparable on the first (*t*_19_ = 3.21, *p* = .00458, BF = 9.88) and the second response (*t*_19_ = 2.92, *p* = .00876, BF = 5.72). There was a modest trend toward an interaction when comparing neutral with uncued items (main effect of cue: *F*_1,19_ = 12.83, *p* = .00199, η_p_^2^ = 0.40, BF = 5.04, main effect of order: *F*_1,19_ = 65.21, *p* = 1.46 × 10^−7^, η_p_^2^ = 0.77, BF = 2.19 × 10^4^, order by cue interaction *F*_1,19_ = 3.027, *p* = .0981, η_p_^2^ = 0.14, BF = 1.53). Although there were noticeable cueing costs during the first response (*t*_19_ = −3.239, *p* = .00432, BF = 10.4), there were none during the second response (*t*_19_ = −1.15, *p* = .264, BF = 0.415).

In sum, the results of Experiment 3 confirmed those of the first two experiments. Recall of cued items was significantly more accurate at no great cost to uncued items. Trial-by-trial correlations also showed no evidence for tradeoffs in accuracy (see Supplementary Results). Interestingly, recall of uncued items was biased away from an item that was cued as relevant but had not yet been recalled, particularly when the order of recall was not known in advance.

## Discussion

This study examined how cueing attention toward an item in working memory affects recall of the cued item as well as recall of the uncued items. Specifically, the study sets out to test whether memory for a cued item improves because it receives a larger portion of a shared memory resource, causing uncued items to lose resources as a consequence. Observers recalled two out of four items, one of which was sometimes cued during the memory delay. There was a robust recall benefit for the cued item, even when a second, uncued item was recalled first. Importantly, there was little evidence for a cost for uncued items. Most strikingly, if the cued item was probed first, the other uncued items were largely recalled with the same accuracy as observed on neutral trials. In addition, there was no evidence of negative correlations in accuracy across trials (i.e., recalling a cued item with higher accuracy did not decrease accuracy for the uncued item on that trial). This cueing benefit without strong, correlated cost equates to a net increase in working memory performance. To the best of our knowledge, no other studies have tested for trial-by-trial trade-offs in memory resources or how these may be affected by cueing, although studies have found that when several features are remembered for each item (color and orientation, e.g.), errors in recalling different features of the same item are uncorrelated (Bays, Wu, & Husain, 2011; Fougnie & Alvarez, 2011). This finding seems inconsistent with a single, finite memory resource that is distributed among all items, because a finite resource account predicts a trade-off in memory between cued and uncued items.

In contrast to single-probe tasks, prompting for multiple responses can test for residual memory of uncued items while equating probe anticipation. After the first response has been made, whether that first response probed a neutral item or a cued item, the second response probed one of the three remaining (uncued) items. Therefore, probe expectation in both conditions was equal. It also reduces strategic variability across participants: Participants retained information about previously uncued items, suggesting that information about those items was not lost as a matter of course. In studies probing a single item, it could be strategic or inconsequential to forget uncued items, deriving some cueing benefit from the freed-up space in memory (Williams et al., 2013; Wolff, Jochim, Akyürek, & Stokes, 2017). In contrast, using multiple cues, we show that benefits can also arise without the need to remove or impoverish uncued information in memory. Other details of our task design also reduce the likelihood of interference effects accounting for the retrocueing benefit. Our tasks included a backward mask after stimulus presentation but before the cue and used a minimally disruptive probe stimulus. Given that, under these circumstances, costs disappeared in Experiment 2, benefits without costs presumably arose from a change in the representational format of the cued item that was independent of the maintenance of other items. As a consequence, there is likely more than one resource pool for the maintenance and prioritization of information in WM, as suggested by several groups (Cowan, 2000; Olivers et al., 2011; Oberauer, 2013). Similar accounts of the benefits of attention in the perceptual domain have been made in the past (Kinchla, Chen, & Evert, 1995). Previous work has proposed a computational model to account for retrocueing benefits which depends on the redistribution of resources to the cued representation (Souza, Rerko, Lin, & Oberauer, 2014). This work found that the trade-off assumption was in good agreement with their data. However, in their model differences in probe anticipation were not accounted for. It will be interesting to see how probe anticipation and memory resource redistribution can be incorporated into a single model.

To our knowledge, only one other study has tested memory for other items by probing more than one item in the recall phase (Rerko et al., 2014). The study found, in two experiments, significant costs to recalling an uncued item *after* having recalled a cued item. Two differences in task design could account for the discrepant findings. First, Rerko and colleagues used six items per array, exceeding the average storage capacity of WM, whereas we used four items. When operating above the capacity limit, the difficulty of continued maintenance of all five uncued items might increase the likelihood of their sudden forgetting (Zhang & Luck, 2009). Also, for larger arrays, the value of remembering any individual uncued item diminishes, so strategic differences might have led observers to accept performance costs for larger arrays. Finally, in Rerko and colleagues’ paradigm, mismatch probes were sometimes items from an unprobed location in the array. Possibly, the increased load could have led to swap errors on uncued trials attributable to a reduction in spatial resolution of feature-location bindings (Rerko et al., 2014). Given the significant costs to uncued items, Rerko and colleagues put forth several explanations for retrocueing benefits: first, uncued items could be (partially) removed from the central part of WM, reducing interference with the cued item and leading to benefits and costs (see also Souza, Rerko, & Oberauer, 2014). Alternatively, benefits could be the result of moving the cued item into the focus of attention, a functional state that singles out one item in WM as the input to the next (cognitive) action (Rerko et al., 2014; Oberauer, 2013). Our experiments show that it is possible in some circumstances to see benefits without costs, emphasizing that the latter explanation is a likely mechanism, at least under some circumstances. Nevertheless, strategic forgetting of uncued memories is also likely to be a beneficial and ecologically valid strategy in many other cases (Williams et al., 2013; Wolff et al., 2017).

The cost–benefit analysis suggested that benefits might arise from transferring cued items to a different functional state (i.e., the focus of attention). Intriguingly, we found novel evidence that bears on how this functional state might operate: when an uncued item was probed unexpectedly, recall was biased away from the cued orientation. The biasing effect in Experiments 1 and 3 might have been the result of competing representations crowding into the focus of attention. When observers did not know when the cued item would be probed, and an uncued item was probed first, then recall was biased away from the cued orientation (E1, E3). Interestingly, knowing the order of recall (in Experiment 2) seemed to eliminate the bias, or at least reduce it (in Experiment 3). Therefore, participants seem to be able to use temporal or sequential information to compartmentalize the recall process, and to control when important items are placed into the focus of attention. Critically, this repulsion effect cannot result from mistakenly reporting the cued item. If the cued feature had been guiding behavior, responses should have been biased *toward* it. Instead, responses were biased away from the cued feature. This kind of interference with recall for uncued items goes against a simpler account where the retrocued item is simply the most likely to be recalled next (as suggested by Souza, Rerko, & Oberauer, 2014). The bias was not present in other cueing conditions.

The repulsive bias could have occurred for two reasons. For one, the appearance of an uncued probe might have prompted observers to remove the representation of the cued orientation from the focus of attention, leading to temporary suppression of and a consequent bias away from the cued feature. Alternatively, the concurrent representation of two response rules during recall of the uncued item might only be possible if they are orthogonalized to some degree. Behavioral and neuroimaging studies of visual attention have argued that orthogonalization of search templates and visual distractors is an optimal strategy for the detection of targets, given a known context of distractors (Navalpakkam & Itti, 2007; Scolari & Serences, 2009; Scolari, Byers, & Serences, 2012). Similar retroactive orthogonalization has been shown to occur in recall from visual WM (Kang & Choi, 2015), although it is unclear whether this is governed by a similar mechanism. Interestingly, in hippocampal long-term memory recall, orthogonalization is likely required for successful read-out of separable patterns (Kohonen & Oja, 1976; Leutgeb, Leutgeb, Treves, Moser, & Moser, 2004; Marr, 1971; McNaughton & Morris, 1987; and possibly also in WM, see Goldman, 2009). Pattern separation may additionally help avoid the merging of the cued and uncued features at the output stage, or the accidental recall of the cued feature. These speculations on the functional purpose of the response bias could be tested more thoroughly with neuroimaging methods.

In sum, this study adds critical behavioral evidence to an evolving picture of WM as a dynamic, multistore architecture (see also Cowan, 2000; D’Esposito & Postle, 2015; Oberauer, 2013; Pasternak & Greenlee, 2005; Sligte, Scholte, & Lamme, 2008). In this framework, WM can be represented in multiple states of prioritization. Cues appear to change the functional state of representations, without necessarily withdrawing resources from other items to do so. One possibility is that cued representations occupy the focus of attention, a state that is more apt to guide behavior. Whether this state change occurs within a single neural circuit (Lewis-Peacock, Drysdale, Oberauer, & Postle, 2012; Oberauer, 2013; Zokaei et al., 2014), or is accomplished by transferring a representation to a different brain area, remains to be tested.

## Supplementary Material

10.1037/xhp0000449.supp
