# Supplementary material for: Benefits of Flexible Prioritization in Working Memory Can Arise Without Costs
Source: J Exp Psychol Hum Percept Perform. 2017 Aug 17;44(3):398–411. doi: 10.1037/xhp0000449 (PMC5868459; doi:10.1037/xhp0000449)
Supplement: Supplementary file 1 [file zfn999173673so1.docx]

**Supplemental Materials**

**Benefits of Flexible Prioritization in Working Memory Can Arise Without Costs**

**by N. E. Myers et al., 2017, *Journal of Experimental Psychology: Human Perception and Performance***

**http://dx.doi.org/10.1037/xhp0000449**

**Supplementary Results**

These analyses are added to give a more complete picture of the results reported in the main text.

**Experiment 1**

*Mixture Model Results*

For completeness and comparison to previous studies (e.g., Murray et al., 2013; Williams et al., 2013; Myers et al., 2015; Wallis et al., 2015), we fit a mixture model to the distribution of response errors. The model allowed us to estimate separately the likelihood of recalling the correct orientation (recall rate) and the precision of remembered orientations (measured by the concentration parameter of the von Mises distribution). The pattern of recall rates across conditions was very similar to the accuracy results reported in the main text. There were main effects of cueing (Fig. S1, left panel, F_2,46_=34.14, p=8.15*10^-10^, η_p_^2^=0.60, BF=1.66*10^8^) and of response order (F_1,23_=36.66, p=3.56*10^-6^, η_p_^2^=0.61, BF=814), with a significant interaction (F_2,36_=5.87, p=0.005, η_p_^2^=0.20, BF=5.92). Cues improved recall rates for both responses (main effect in 2x2 ANOVA: F_1,23_=25.52, p=4.10*10^-5^, η_p_^2^=0.53, BF=8.72*10^3^, cueing benefits during the first response: t_23_=2.65, p=0.014, BF=3.60, second response: t_23_=5.62, p=1.02*10^-5^, BF=2.12*10^3^). Although recall rates, in general, decreased for the second response (main effect of order, F_1,23_=57.83, p=1.01*10^-7^, η_p_^2^=0.72, BF=636), the cueing benefit increased (interaction with probe order: F_1,23_=15.87, p=0.00059, η_p_^2^=0.41, BF=8.78*10^3^).

In addition to the cueing benefits, there was also a significant reduction in recall rate when uncued items were probed (main effect of cue, F_1,23_=10.29, p=0.0039, η_p_^2^=0.31, BF=1.26, main effect of order, F_1,23_=29.66, p=1.55*10^-5^, η_p_^2^=0.56, BF=3.28*10^4^). This was caused by a significant reduction in recall rate during the first response (t_23_=-4.18, p=0.00036, BF=86.7). By contrast, there was no reduction in recall rate when uncued items were probed second (t_23_=0.078, p=0.94, BF=0.215).

Although our focus lay on recall rate, similar effects were also visible in the precision parameter of the mixture model (Fig. S1, right panel, main effect of cue: F_2,46_=18.34, p=1.39*10^-6^, η_p_^2^=0.44, BF=2.24*10^4^, main effect of order: F_1,23_=36.35, p=3.79*10^-6^, η_p_^2^=0.61, BF=4.39*10^4^, but no interaction: F_2,46_=1.76, p=0.184, η_p_^2^=0.07, BF=0.328). As with recall rate, cued items were recalled with significantly higher precision than neutral items (first response: t_23_=3.58, p=0.0016, BF=23.3, second response: t_23_=3.21, p=0.0039, BF=10.9), whereas uncued items only carried a modest precision cost during the first response (t_23_=-2.17, p=0.041, BF=1.52), but none during the second (t_23_=-0.62, p=0.542, BF=0.256).


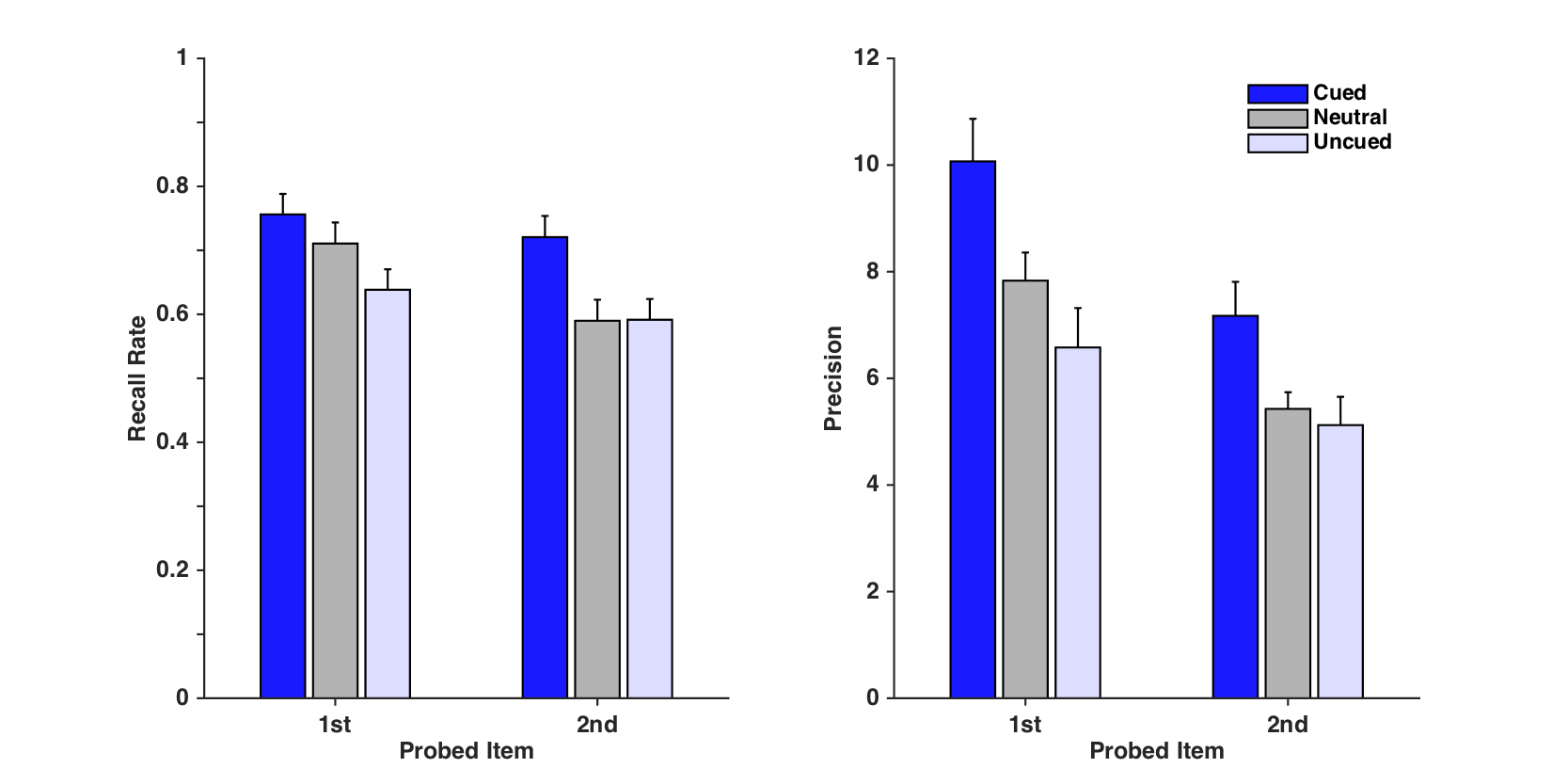


*Figure S1. Mixture modeling results in Experiment 1. Left panel. Recall rate was modulated both by cue type and by probe order. Error bars denote within-observer standard error of the mean. Right panel. Precision parameter estimates for the same conditions.*

*Between-observer correlations*

While there was no evidence of a trade-off across trials (in any of the conditions), there could have been a trade-off between benefits and costs across participants. This would manifest as a negative correlation between the cueing benefits (cued vs. neutral performance) and the costs to uncued items (uncued vs. neutral performance) – the bigger the benefit, the larger the costs. This is not what we saw. In fact, when testing for correlations across observers, we found that cueing benefits and costs were largely uncorrelated (p>0.17), with the exception of a significant *positive* correlation between cued-vs.-neutral and uncued-vs.-neutral accuracy for the second response (Pearson r_23_=+0.505, p=0.012). In other words, participants with large cueing benefits tended to have smaller costs to the uncued item. There was also a trend towards a positive correlation between cueing benefits on the first and second responses (r_23_=+0.373, p=0.073).

*Response bias during the second response*

Unlike response 1 (see main text), the second response was consistently biased away from the item recalled first (Fig. S2, average across conditions, t_23_=-3.56, p=0.00168, BF=22.27), with no main effect of condition (F_2,46_=2.33, p=0.108, η_p_^2^=0.09, BF=0.755). Nonetheless, the biasing effect was statistically significant only in the cue conditions (cued: t_23_=-3.30, p=0.0032, BF=12.90, uncued: t_23_=-3.13, p=0.0047, BF=9.17, neutral: t_23_=-1.21, p=0.237, BF=0.413).


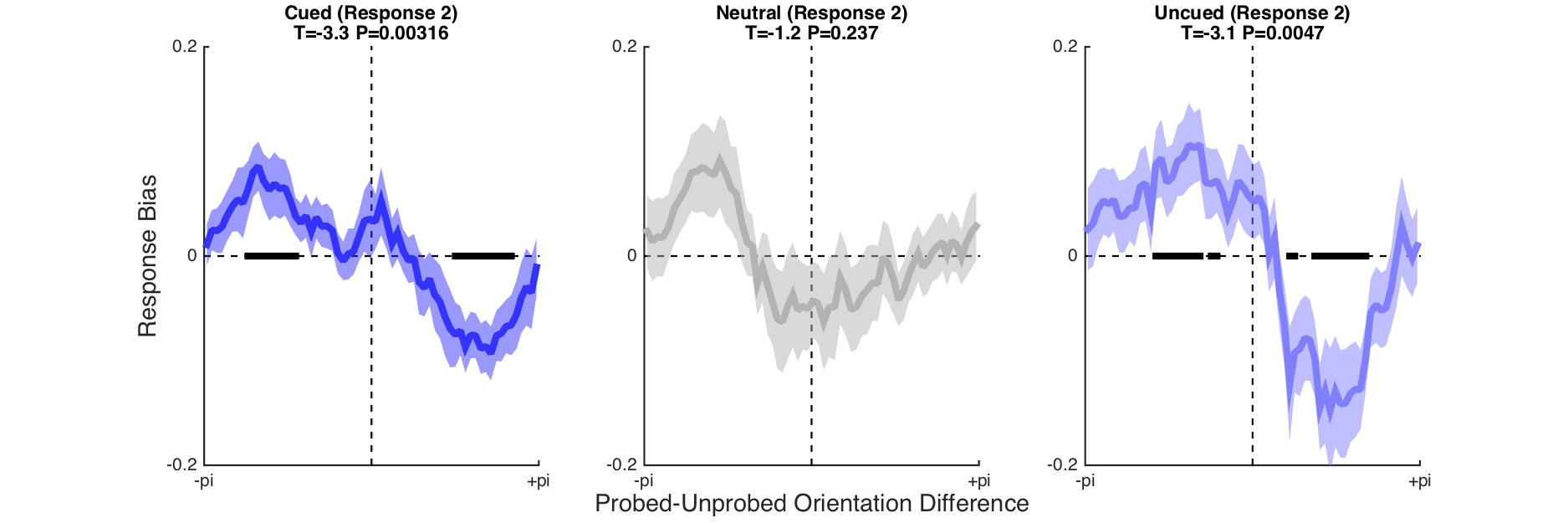


*Figure S2. Response bias during the second of two responses, with respect to the orientation of the item that was probed first. Conventions are the same as in Fig. 4. Generally, responses were biased away from the orientation of the first probed item.*

**Experiment 2**

*Mixture-modeling results*

As in experiment 1, there was a significant main effect of cue type on recall rates (Fig. S3, left panel, F_2,46_=18.62, p=1.19*10^-6^, η_p_^2^=0.45, BF=1.21*10^6^) and of order (F_1,23_=87.28, p=2.71*10^-9^, η_p_^2^=0.79, BF=1.66*10^4^), with no significant interaction (F_2,46_=0.24, p=0.791, η_p_^2^=0.01, BF=0.133). Retrocues increased the recall rate on both the first (t_23_=5.33, p=2.05*10^-5^, BF=1.13*10^3^) and second response (t_23_=3.32, p=0.00298, BF=13.6). Conversely, uncued items did not suffer significant reductions in recall rate (main effect of cue: F_1,23_=2.04, p=0.167, η_p_^2^=0.08, BF=0.392, first response: t_23_=-1.35, p=0.19, BF=0.480; second response: t_23_=-0.64, p=0.53, BF=0.258). Again, this analysis confirmed an asymmetric pattern of significant benefits at no cost (especially, and crucially, during the second response). Additionally, it appears that recall rates for uncued items did not suffer for either response when cues indicated the recall order.


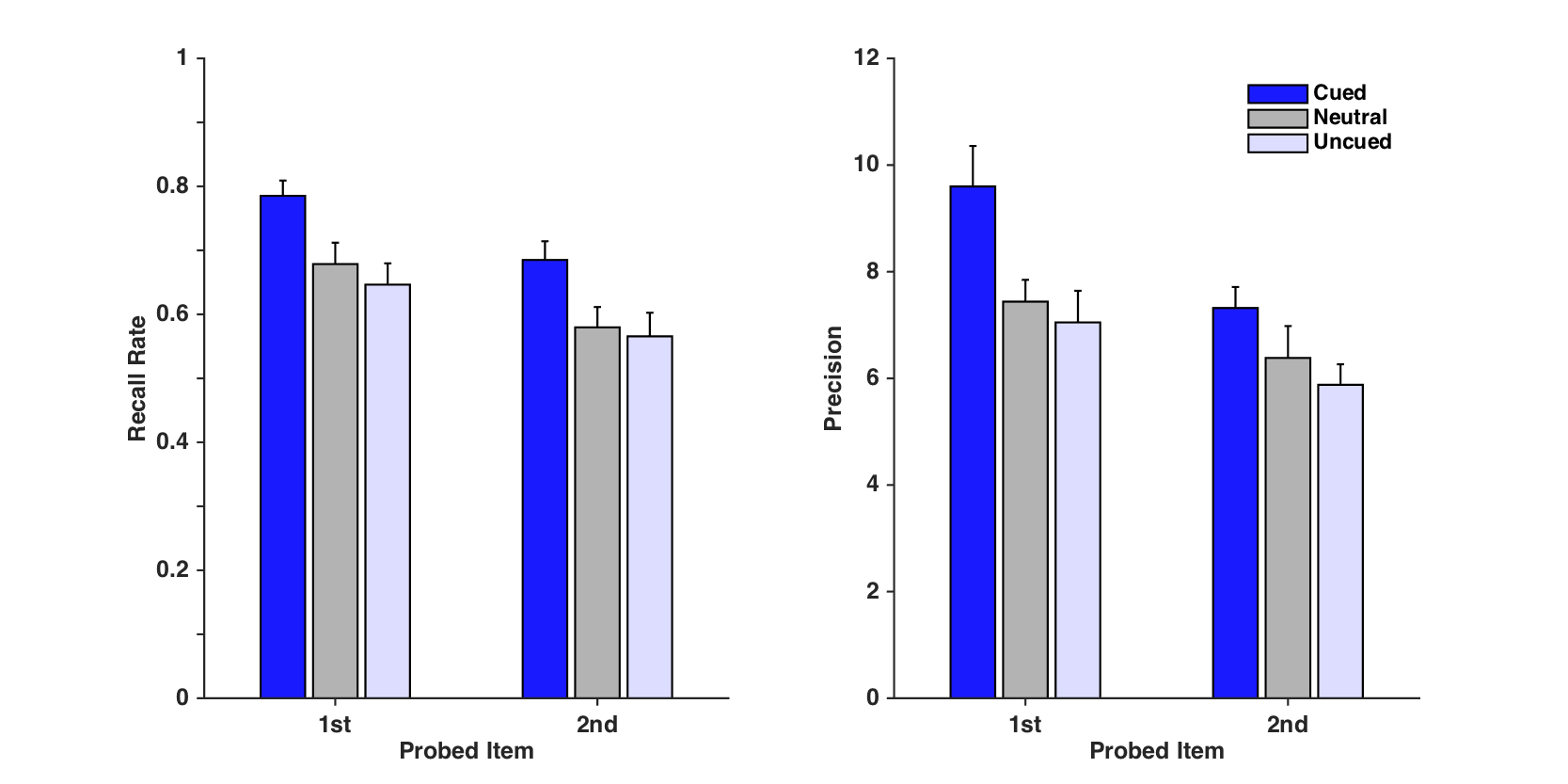


*Figure S3. Mixture modeling results in Experiment 2. Left panel. Recall rate was modulated both by cue type and by probe order. Importantly, during the second response, uncued and neutral items had equivalent recall rates, indicating that there are no lingering costs to improving the accuracy of a retrocued item. Error bars denote within-observer standard error of the mean. Right panel. Precision parameter estimates (from the mixture model fit) for the same conditions.*

Regarding the precision parameter of the model fit, there were main effects of cueing (Fig. S3, right panel, F_2,46_=12.20, p=5.61*10^-5^, η_p_^2^=0.35, BF=473) and of recall order (F_1,23_=23.81, p=6.30*10^-5^, η_p_^2^=0.51, BF=216), with no significant interaction (F_2,46_=1.23, p=0.303, η_p_^2^=0.05, BF=0.322). However, this pattern broke down into a significant increase in precision for the cued item on the first response (t_23_=3.17, p=0.0043, BF=9.87), with no strong effects on fidelity for the cued item on the second response (t_23_=1.74, p=0.095, BF=0.795), and no significant costs to uncued items (first response: t_23_=-0.60, p=0.556, BF=0.253, second response: t_23_=-0.99, p=0.328, BF=0.336).

*Between-observer correlations*

There were no significant correlations across observers between cueing benefits (accuracy on cued minus neutral trials) and costs (uncued minus neutral, all pairwise p>0.16, all r<0.30). The only significant correlation was between cueing benefits on the first and the second responses (Pearson r_23_=+0.59, p=0.0024).

*Response bias during the second response*

In experiment 2 the responses to the second probe were not strongly biased away from the first probed item (average across conditions, t_23_=-1.57, p=0.130, BF=0.629), with no main effect of condition (F_2,46_=0.95, p=0.39, η_p_^2^=0.04, BF=0.240). While the biasing effect was not significant for uncued and neutral trials (uncued: t_23_=-0.836, p=0.412, BF=0.294, neutral: t_23_=-0.652, p=0.521, BF=0.260), there was a significant repulsive bias for cued items (t_23_=-3.987, p=0.000581, BF=56.5).

**Experiment 2b**


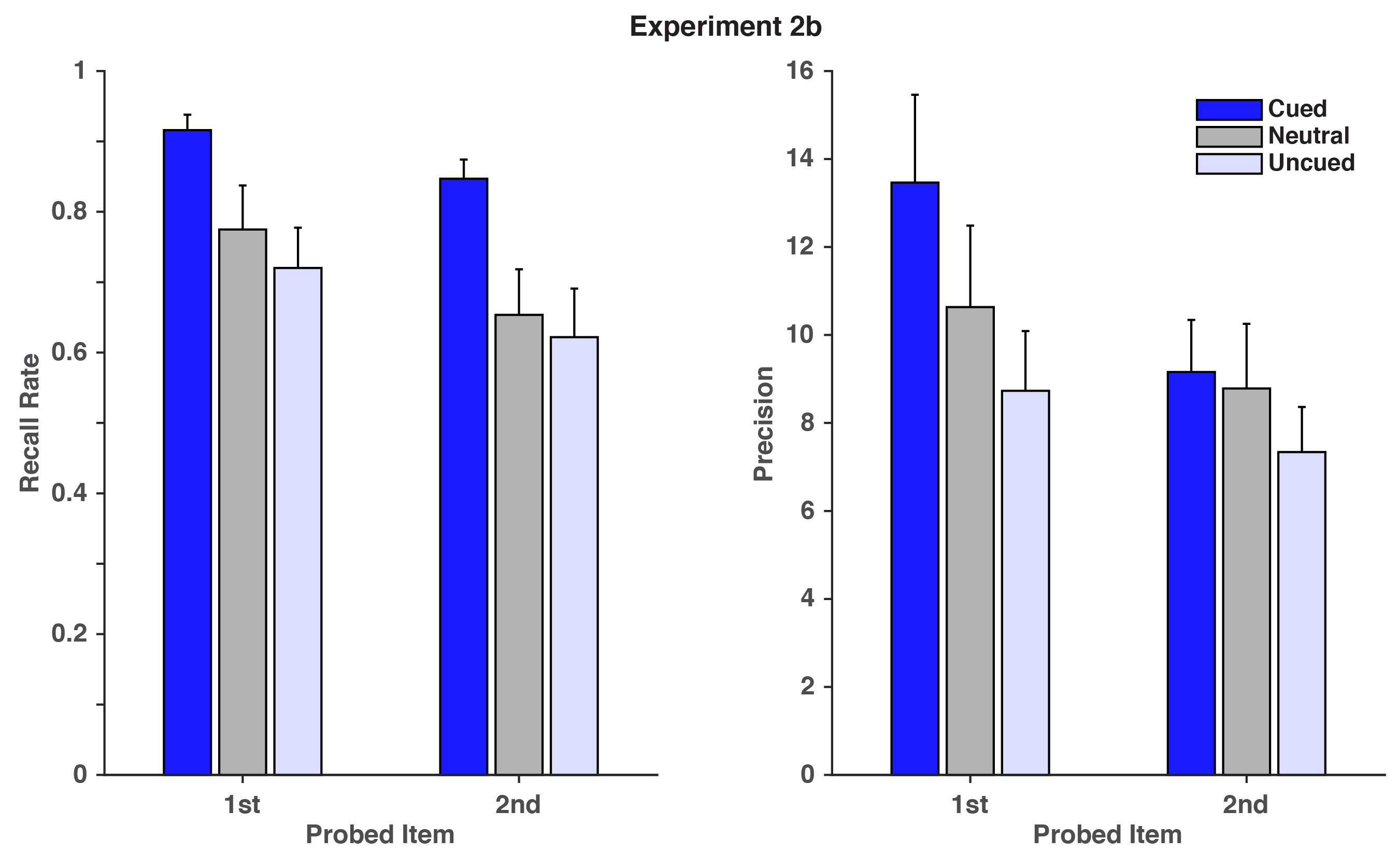


*Figure S4. Mixture modeling results in Experiment 2b (order cued, as in Experiment 2). Error bars denote within-observer standard error of the mean.*

*Accuracy*

There were strong cueing effects on accuracy (Fig. 4, 2x3 ANOVA, main effect of cue: F_2,30_=42.46, p=1.78*10^-9^, η_p_^2^=0.74, BF=1.68*10^6^), as well as a significant effect of response order (F_1,15_=19.04, p=5.56*10^-4^, η_p_^2^=0.56, BF=1027) and an inconclusive interaction (F_2,30_=3.96, p=0.029, η_p_^2^=0.21, BF=0.64). An ANOVA using only cued and neutral responses (to test for benefits independent of costs) also showed an effect of cueing (2x2 ANOVA, main effect of cue: F_1,15_=37.58, p=1.92*10^-5^, η_p_^2^=0.71, BF=594), and also a main effect of order (F_1,15_=15.55, p=0.0013, η_p_^2^=0.51, BF=141). There was no cue-by-order interaction (F_1,15_=0.54, p=0.474, η_p_^2^=0.03, BF=0.347), indicating that cueing benefits were comparable on the first (t_15_=5.775, p=3.66*10^-5^, BF=694) and the second response (t_15_=4.91, p=1.88*10^-4^, BF=164).

Analysis of cueing costs, by comparing neutral to uncued items, revealed a modest interaction between cue and response (order by cue interaction F_1,15_=4.56, p=0.050, η_p_^2^=0.23, BF=1.46, main effect of cue: F_1,15_=9.96, p=0.0065, η_p_^2^=0.40, BF=0.39, main effect of order: F_1,15_=18.7, p=5.9*10^-4^, η_p_^2^=0.56, BF=1.61*10^4^), which was reflected also in the significant cueing costs during the first response (t_15_=-3.71, p=0.002, BF=20), with no cueing costs during the second response (t_15_=0.53, p=0.606, BF=0.29). The absence of cueing costs during response 2 confirms the finding from experiment 2: robust cueing benefits can be observed at no cost to uncued items. Nevertheless, the significant costs during response 1, even when order was cued, indicate that performance on uncued items may still be worse when a different item is in a prioritized state.

*Mixture-Modeling Results*

As in experiments 1 and 2, there was a significant main effect of cue type on recall rates (Fig. S4, F_2,30_=13.89, p=5.37*10^-5^, η_p_^2^=0.48, BF=1.99*10^6^) and of order (F_1,15_=29.19, p=7.33*10^-5^, η_p_^2^=0.66, BF=12.8), with a marginally significant interaction (F_2,30_=3.29, p=0.051, η_p_^2^=0.18, BF=0.207). Retrocues increased the recall rate on both the first (t_15_=2.64, p=0.0186, BF=3.30) and second response (t_15_=3.67, p=0.00228, BF=18.9). Conversely, uncued items only resulted in significant reductions in recall rate during the first response (first response: t_15_=-3.82, p=0.0017, BF=24.4; second response: t_15_=-1.56, p=0.14, BF=0.702; main effect of cue: F_1,15_=8.57, p=0.010, η_p_^2^=0.36, BF=1.10; interaction: F_1,15_=1.47, p=0.244, η_p_^2^=0.09, BF=0.396).

The precision parameter of the model fit also showed main effects of cueing (F_2,30_=4.82, p=0.0154, η_p_^2^=0.24, BF=5.95) and of recall order (F_1,15_=7.93, p=0.013, η_p_^2^=0.35, BF=11.6), with no significant interaction (F_2,30_=2.43, p=0.105, η_p_^2^=0.14, BF=0.431). As in other experiments, there was a small but significant increase in precision for the cued item on the first response (t_15_=2.47, p=0.0258, BF=2.53), with no strong effects on fidelity for the cued item on the second response (t_15_=0.24, p=0.813, BF=0.262), and no significant costs to uncued items (first response: t_15_=-1.48, p=0.158, BF=0.639, second response: t_15_=-0.95, p=0.359, BF=0.376).

*Trial-by-trial correlations*

In Experiment 2b, trialwise correlations in accuracy where either absent or positive, indicating no tradeoff between the two responses. We saw numerically positive but small correlations for all three cueing conditions (mean±s.e.m. Pearson r for neutral: 0.093±0.027, t_15_=3.40, p=0.0039, BF=11.9, cued first: 0.046±0.025, t_15_=1.85, p=0.0837, BF=1.02, cued second: 0.020±0.028, t_15_=0.72, p=0.485, BF=0.320), with no conclusive effect of cue type on the correlation (F_2,30_=2.49, p=0.100, η_p_^2^=0.14, BF=0.99). This again contradicts the hypothesis of any trade-off across trials.

*Between-observer correlations*

There were no significant correlations across observers between cueing benefits (accuracy on cued minus neutral trials) and costs (uncued minus neutral, all pairwise p>0.47, all r<0.19). There were significant correlations between cueing benefits on the first and the second responses (Pearson r_15_=+0.52, p=0.041) and between cueing costs on the first and second responses (Pearson r_15_=-0.65, p=0.006).

*Response bias*

As in experiment 2, responses were not significantly biased during the first probe (average across conditions, t_15_=1.06, p=0.306, BF=0.413, main effect of condition: F_2,30_=1.64, p=0.21, η_p_^2^=0.10, BF=0.508, p>0.21 for individual cueing conditions). Similarly, there was no strong bias during the second response (average across conditions, t_15_=0.34, p=0.740, BF=0.269), with no main effect of condition (F_2,30_=1.72, p=0.197, η_p_^2^=0.10, BF=0.519). On the second response, the biasing effect was not significant for uncued and neutral trials (uncued: t_15_=0.362, p=0.722, BF=0.272, neutral: t_15_=0.881, p=0.392, BF=0.356), but there was a significant repulsive bias for cued items (t_15_=-3.647, p=0.00238, BF=18).

**Experiment 3**

*Probe order not cued (as in E1)*


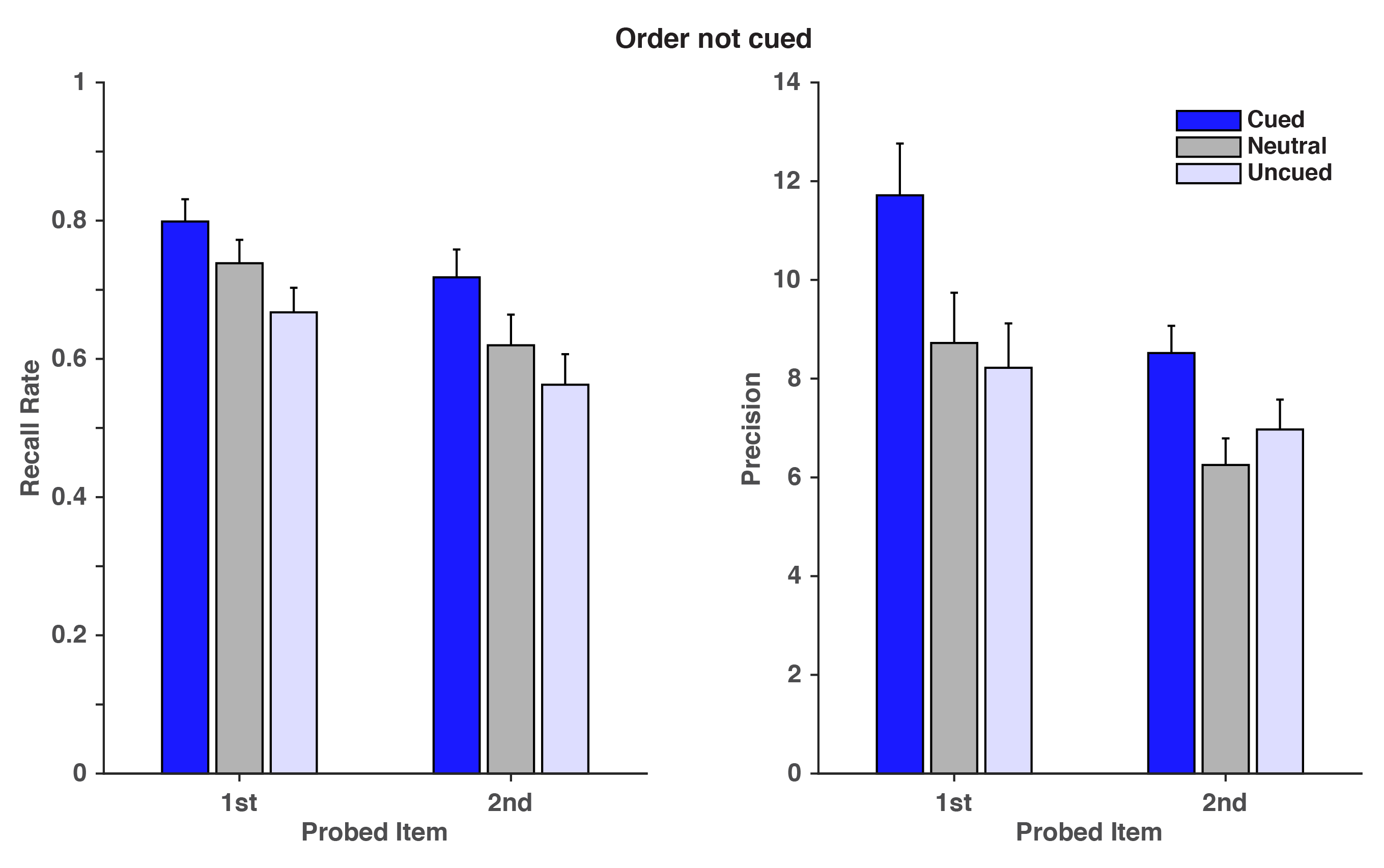


*Figure S5.Mixture modeling results in Experiment 3 (order not cued, as in Experiment 1). Left panel. Recall rate estimated separately per condition. Right panel. Precision parameter estimates from the same model fit.*

*Mixture-Modeling Results*

Given the additional factor in Experiment 3 (cue order), the number of trials per condition was reduced from 160 to 100 trials, which may be at the low end of what is necessary to fit reliably the two-parameter mixture model. Therefore, these model estimates should be treated somewhat more cautiously. As before, we focused on the effects of cueing on the recall rate. Again there was a significant main effect of cue type (F_2,38_=12.54, p=6.59*10^-5^, η_p_^2^=0.40, BF=5.09*10^3^) and of order (F_1,19_=26.89, p=5.26*10^-5^, η_p_^2^=0.59, BF=548), with no significant interaction (F_2,38_=0.46, p=0.635, η_p_^2^=0.02, BF=0.162). Retrocues increased the recall rate on both the first (t_19_=2.90, p=0.0092, BF=5.50) and, to some extent, on the second response (t_19_=2.42, p=0.0259, BF=2.34). Conversely, uncued items did not lead to significant reductions in recall rate (first response: t_19_=-1.83, p=0.083, BF=0.945; second response: t_19_=-1.52, p=0.145, BF=0.624), although there was a marginally significant main effect of cue (F_1,19_=4.72, p=0.043, η_p_^2^=0.20, BF=0.925). Again, this analysis confirmed the same asymmetric pattern of significant benefits at no or low costs that was seen in experiments 1 and 2.

As before, the pattern of recall rates was also reflected in the cueing effects on precision. There were main effects of cueing (F_2,38_=22.74, p=3.21*10^-7^, η_p_^2^=0.54, BF=78.6) and of recall order (F_1,19_=11.05, p=0.00357, η_p_^2^=0.37, BF=6805), with no significant interaction (F_2,38_=1.73, p=0.191, η_p_^2^=0.08, BF=0.289). However, this pattern broke down into a significant increase in fidelity for the cued item (first response: t_19_=4.85, p=0.00011, BF=254.9, second response: t_19_=2.63, p=0.0166, BF=3.37), with no significant costs to uncued items (first response: t_19_=-1.23, p=0.236, BF=0.447, second response: t_19_=0.16, p=0.872, BF=0.235).

*Probe order cued (as in E2)*


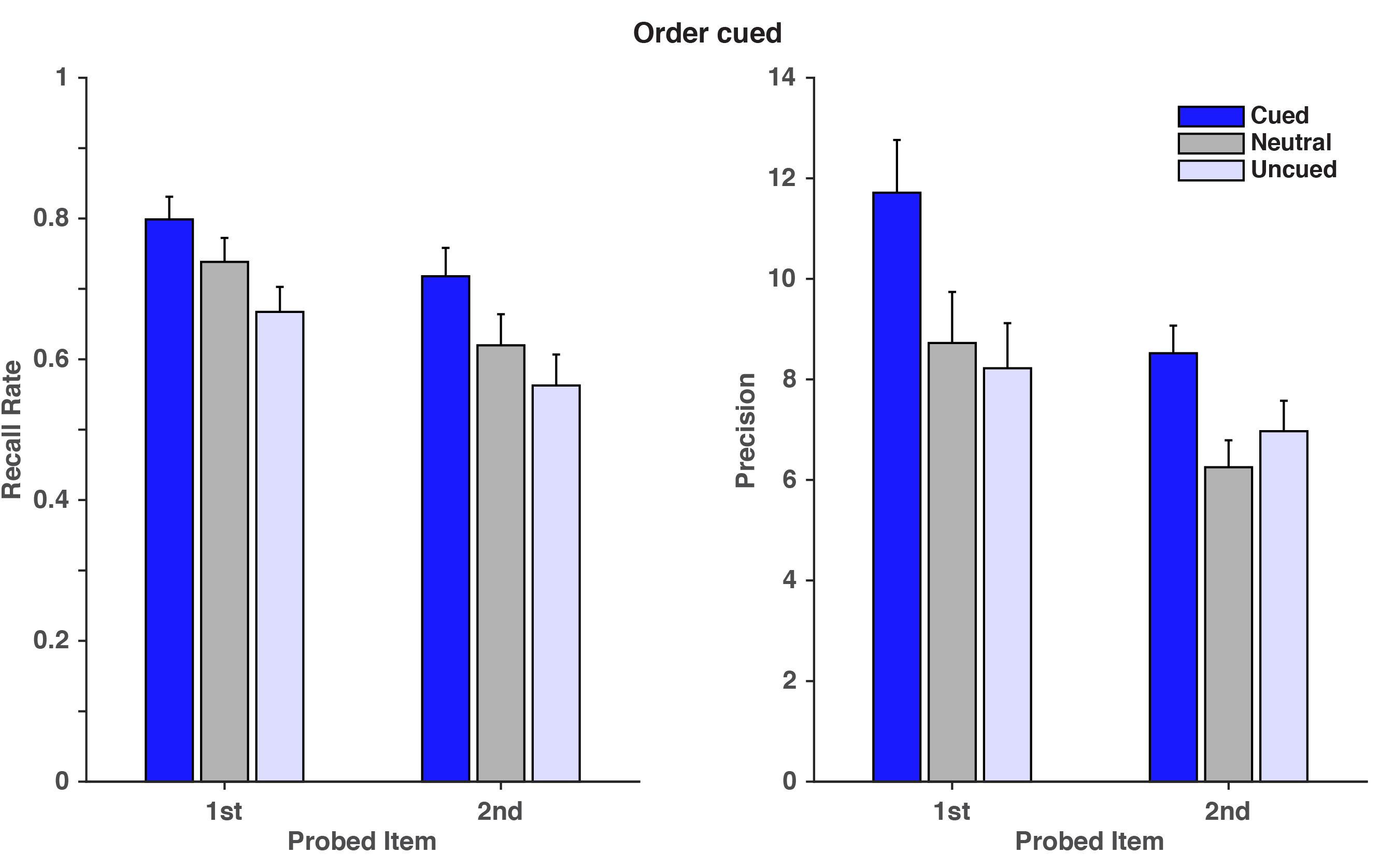


*Figure S6. Mixture modeling results in Experiment 3 (order cued, as in Experiment 2). Left panel. Recall rate estimated separately per condition. Right panel. Precision parameter estimates from the same model fit.*

*Mixture-Modeling Results*

There was a significant main effect of cue type (F_2,38_=23.38, p=2.40*10^-7^, η_p_^2^=0.55, BF=3.36*10^5^) and of order (F_1,19_=49.53, p=1.06*10^-6^, η_p_^2^=0.72, BF=1.19*10^5^), with no significant interaction (F_2,38_=0.48, p=0.621, η_p_^2^=0.02, BF=0.167). Retrocues increased the recall rate on both the first (t_19_=3.01, p=0.0072, BF=6.78) and second response (t_19_=2.72, p=0.0137, BF=3.95). Unlike in the other experiments, there was a significant reduction in recall rate in uncued compared to neutral trials for both responses (main effect of cue: F_1,19_=13.59, p=0.00157, η_p_^2^=0.42, BF=14.0; first response: t_19_=-3.17, p=0.0051, BF=9.08; second response: t_19_=-2.50, p=0.0219, BF=2.68). This relatively modest cost to the recall rate for the second response contradicts the absence of cueing costs on accuracy for the same condition (p=0.26, see above). One possibility is that the significant reduction in recall rate could be partially caused by inaccuracies in the parameter estimation of the mixture model. This possibility is partially reflected in a numerical increase in the precision parameter on uncued trials (precision on uncued trials, second response: 7.78±0.78, neutral trials: 6.56±0.775, t_19_=+1.223, p=0.236, BF=0.446), which was not seen during the first response (uncued versus neutral trials: t_19_=-1.31, p=0.205, BF=0.490, leading to a cue-by-order interaction: F_1,19_=5.96, p=0.0246, η_p_^2^=0.24, BF=1.29). Furthermore, there was a significant negative correlation between cueing costs to uncued items on recall rate and cueing benefits to the same uncued items on precision (Pearson r=-0.626, p=0.0032), indicating the possibility of a trade-off between the parameters when the mixture model was fit.

In addition, precision was increased on cued, compared to neutral, trials (main effect of cue: F_1,19_=14.16, p=0.0013, η_p_^2^=0.43, BF=44.6, first response: t_19_=2.84, p=0.011, BF=4.91, second response: t_19_=2.50, p=0.0218, BF=2.69).

*Trial-by-trial correlations*

As with experiments 1 and 2, there was no evidence for trial-by-trial tradeoffs in accuracy. Instead, we again saw modest but significantly *positive* correlations for all three cueing conditions, both when the probe order was cued (mean±s.e.m. Pearson r for neutral: 0.076±0.020, t_19_=3.77, p=0.0013, BF=29.2, cued first: 0.049±0.024, t_19_=2.02, p=0.057, BF=1.25, cued second: 0.069±0.027, t_19_=2.53, p=0.020, BF=2.87) and when order was not cued (neutral: 0.054±0.020, t_19_=2.64, p=0.016, BF=3.44, cued first: 0.059±0.021, t_19_=2.86, p=0.01, BF=5.10, cued second: 0.082±0.028, t_19_=2.87, p=0.010, BF=5.17). Correlations also did not diminish in magnitude on cued trials compared to neutral trials (order cued, cued first: t_19_=-0.95, p=0.354, BF=0.347, cued second: t_19_=-0.18, p=0.858, BF=0.236, order not cued, cued first: t_19_=0.17, p=0.868, BF=0.235, cued second: t_19_=0.98, p=0.334, BF=0.355).

*Between-observer correlations*

There were no significant correlations across observers between cueing benefits (accuracy on cued minus neutral trials) and costs (uncued minus neutral, all pairwise p>0.32, all r<0.23), with three notable exceptions. When probe order was cued, benefits and costs on the second response were positively correlated (Pearson r_19_=0.56, p=0.011). Also, cueing costs on the first response and cueing benefits on the second response were negatively correlated (Pearson r_19_=-0.72, p=0.0004). This appears to be evidence of a trade-off between benefits and costs. However, this correlation seems to have been driven by two outliers (>2 SD outside the mean for both conditions) – using Spearman correlations, this relationship was reduced to a trend (Spearman’s rho_19_=-0.409, p=0.075). After excluding the two outliers, the Pearson correlation was also no longer significant (r_17_=-0.367, p=0.134). Finally, when probe order was not cued, there was a significant positive correlation between cueing benefits on the first and second responses (Pearson r_19_=+0.523, p=0.018), again contradicting the idea that resource trade-offs drive the retrocueing benefit.

*Response bias during the second response*

In experiment 3, we again examined how recall during the second response was biased by the item recalled first. Overall, responses were again biased away, as in experiment 1 (average across all conditions, t_19_=-3.746, p=0.00137, BF=27.85), with no main effect of condition (F_2,38_=0.96, p=0.391, η_p_^2^=0.05, BF=0.164) or order cue (F_1,19_=0.877, p=0.361, η_p_^2^=0.04, BF=0.368, interaction: F_2,38_=0.26, p=0.777, η_p_^2^=0.01, BF=0.161).


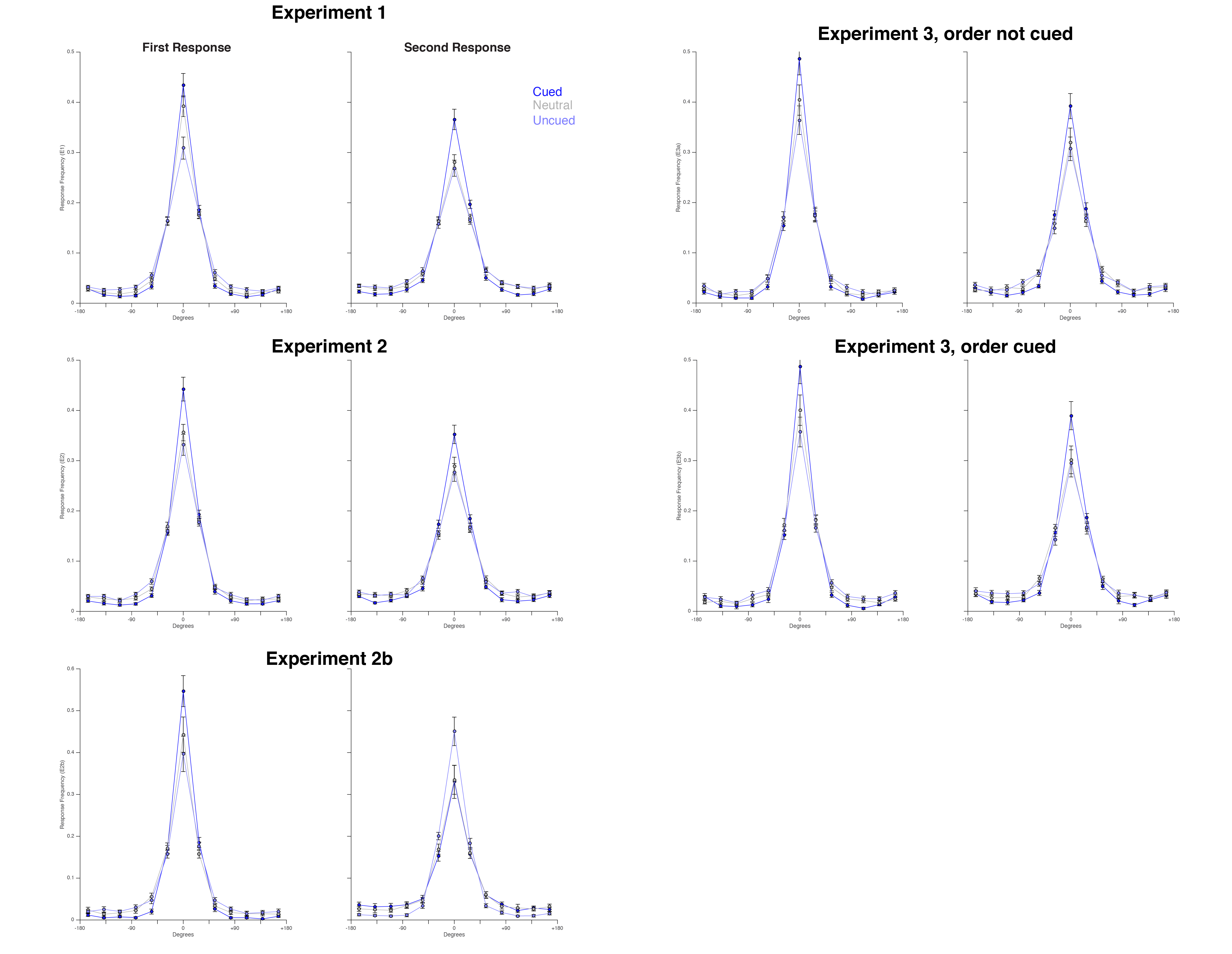


*Figure S7. Response histograms for all experiments. Each plot shows the distribution of errors, centered on the correct value (0º), separately for the cued, neutral, and uncued conditions, and separately for the first and second responses.*
